# Freeze or Forget? Virtual Attack Effects in an Emotional Sternberg Task

**DOI:** 10.5964/ejop.v14i2.1473

**Published:** 2018-06-19

**Authors:** Thomas Edward Gladwin, Matthijs Vink

**Affiliations:** a Department of Psychology and Counseling, University of Chichester, Chichester, United Kingdom; b Department of Psychiatry, Brain Center Rudolf Magnus, Utrecht University Medical Center, Utrecht, The Netherlands; c Departments of Developmental and Experimental Psychology, Utrecht University, Utrecht, The Netherlands; Department of Psychology, Webster University Geneva, Geneva, Switzerland; University of Liverpool, Liverpool, United Kingdom

**Keywords:** Emotional Sternberg, freezing, virtual attack, faces, interference

## Abstract

Emotionally salient stimuli have the ability to disrupt cognitive processing. This kind of disruption involves effects on working memory and may be related to mental health problems. To explore the nature of such emotional interference on working memory, a Virtual Attack Emotional Sternberg Task (VAEST) was used. Neutral faces were presented as distractors and warning signals, which were sometimes followed by a virtual attack, created by having the neutral face turn angry while the image was enlarged. The attack was hypothesized to have one of two effects: to disrupt cognitive processing and thereby increase interference effects, or to terminate a state of freezing and thereby reduce interference effects. The task was successfully completed online by a sample of 59 students. Results clearly show that the virtual attack caused a reduction of interference relative to no-attack trials. The apparent cognitive disruption caused by emotional distractors may thus reflect freezing, which can be reversed by a freeze-terminating stimulus.

Dual-process or dual-system theories posit a distinction between “hot” and “cold” cognitive systems, functions or processes (Metcalfe & Mischel, 1999; Prencipe et al., 2011; Van den Wildenberg & Crone, 2005), using a variety of broadly related terms (Evans, 2008; Gladwin & Figner, 2014; Moors & De Houwer, 2006) such as automatic versus controlled (Schneider & Chein, 2003; Schneider & Shiffrin, 1977) or impulsive versus reflective (Strack & Deutsch, 2004). One kind of process is “hot”, emotional, fast, stimulus-driven and inflexible, and tends to produce impulsive behavior. The other kind of process is “cold”, controlled, effortful, long-term focused, allowing reflective thought and the inhibition of impulses. Theoretical concerns about the validity of a strict separation between opposing hot and cold systems have been raised (Keren, 2013; Keren & Schul, 2009), as, for instance, cognitive processes can have both hot or automatic and cold or reflective attributes (Bargh, 1994). Nevertheless, it does appear to be the case that “hot” emotionally salient stimuli can disrupt cognitive processing necessary for “cold” cognition, for instance by evoking intrusions and rumination which demand cognitive resources (Curci, Lanciano, Soleti, & Rimé, 2013; Curci, Soleti, Lanciano, Doria, & Rimé, 2015).

One source of evidence for such interference is the Emotional Sternberg Task (Sternberg, 1966; Unsworth & Engle, 2007; Wickens, Hyman, Dellinger, Taylor, & Meador, 1986). Trials in the basic Sternberg task consist of three phases: an encoding phase in which participants are presented with a memory set of items; a maintenance phase in which the memory set must be held in working memory (Baddeley, 1992, 2012; Kane & Engle, 2003; Petrides & Baddeley, 1996; Postle, 2006); and a probe phase in which it is tested whether the memory set has been successfully maintained. Variants of the Sternberg task have been developed to tax additional aspects of working memory by presenting distractors during the maintenance period (Unsworth & Engle, 2007; Unsworth, Redick, Heitz, Broadway, & Engle, 2009). This allows disruptive effects of various kinds of stimuli or tasks to be tested. In the emotional Sternberg task, distractors are emotionally salient stimuli; that is, stimuli that draw attention due to their emotional content. Emotionally negative distractors have been shown to cause greater interference, in terms of slower or less accurate responses to probes, than neutral distractors (Dolcos & McCarthy, 2006; Oei, Tollenaar, Elzinga, & Spinhoven, 2010; Oei, Tollenaar, Spinhoven, & Elzinga, 2009). At a neural level, emotional distractors increase activity in regions related to emotion, such as the amygdala and ventrolateral prefrontal cortex, and reduce activity in regions related to working memory, such as dorsolateral prefrontal cortex and parietal cortex (Dolcos & McCarthy, 2006; Oei et al., 2010, 2009). Conversely, increased working memory load decreases activity related to emotional distractors (Erk, Kleczar, & Walter, 2007; Van Dillen, Heslenfeld, & Koole, 2009).

However, the effect of negative emotional stimuli can also be seen as evoking defensive responses such as fight or flight. Previous studies have used emotional stimuli and virtual attacks to induce defensive responses (Gladwin, Hashemi, van Ast, & Roelofs, 2016; Heesink, Gladwin, et al., 2017; Mobbs et al., 2007; Montoya, Van Honk, Bos, & Terburg, 2015; Nieuwenhuys, Savelsbergh, & Oudejans, 2012). Of particular interest to the current study is the defensive response of freezing, a fundamental defensive response that has been argued to be a state involving simultaneous inhibition and preparation for action (Gladwin et al., 2016; Roelofs, 2017). As long as the freeze state persists the organism is highly prepared to “leap into action” and generate fast responses to potentially threatening stimuli (Gladwin et al., 2016; Lojowska, Gladwin, Hermans, & Roelofs, 2015) but is inhibiting action in parallel (Roseberry & Kreitzer, 2017). From this perspective, emotional stimuli could exert effects on performance by evoking a behavioral freeze state rather than by disrupting working memory itself.

To the aim of investigating whether emotional distractors cause "freezing" versus "forgetting", a Virtual Attack Emotional Sternberg Task (VAEST) was developed allowing the study of time-dependent effects of dynamic, threatening distractors on cognitive processing. The distractors in this task consisted of neutral faces that could randomly perform a virtual attack by turning angry and visually zooming in towards the participant. The task thus involved two kinds of interference: that due to the presentation of the neutral face, and that due to the virtual attack. As neutral faces in the task predicted a possible attack, they were expected to evoke this anticipatory freeze and thereby delay responses to probe stimuli of the “cold” task. Attack trials could have two possible effects. If interference effects are due to emotionally salient stimuli capturing processing resources, interference should be greater following an attack. However, attacks could also have the effect of breaking the freeze state, as the anticipated threat has then actually occurred. In that case, interference effects would decrease relative to trials when the attack does not occur, in which situation participants are still in the freeze state when probe stimuli occur. Any such effect could be time-dependent, reflecting the time course of cognitive processes underlying interference, as found previously in an alcohol Sternberg task (Gladwin & Wiers, 2012). The task thus included a manipulation of the time between distractor-events and probe stimuli.

## Method

### Participants

Participants were recruited from a student population and received study credits for completing the study, which was performed online. Participants gave informed consent and the study was approved by the local ethics review board. 62 participants completed the experiment, of which three were rejected due to very low accuracy (below 75% over the whole task, or below 50% on any condition), leaving 59 participants for analysis (48 female, 11 male; mean age 21.3, *SD* = 2.15). Participants were not in treatment for psychiatric or neurological problems.

### Virtual Attack Emotional Sternberg Task (VAEST)

**Figure 1:**
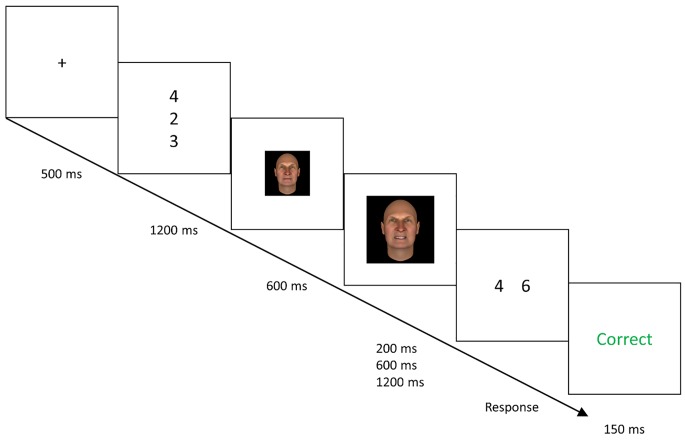
Illustration of the task. *Note.* Illustration of the Virtual Attack Emotional Sternberg Task. Trials consisted of a fixation cross, presentation of the memory set, maintenance period, and probe stimulus. The Figure shows an Attack trial, in which the first 600 ms of the maintenance period contained a neutral face. At 600 ms, the face’s expression turned angry and the face “jumped out at” the participant by increasing in size, suggesting approach. 200, 600 or 1200 ms following the attack, the probe stimulus was presented. The probe remained onscreen until a left-key or right-key response was given, indicating which of the numbers was in the current memory set. Correct answers were followed by a feedback screen briefly showing “Correct” in green, and incorrect answers were followed by “Incorrect” in red. On Neutral Face trials, the attack did not occur and the neutral face remained onscreen until the probe. On such trials, the face remained onscreen for the initial 600 ms plus the varying 200, 600, or 1200 ms. On Null trials, no face appeared, and only a fixation cross was shown during the maintenance period.

Figure 1 shows an illustration of the VAEST (programmed in JavaScript; source code is available on request). Trials began with the presentation of a memory set of three different numbers from 1 to 9, positioned in a vertical column, for 1200 ms. This was followed by a maintenance period of 800, 1200, or 1800 ms; the duration was selected randomly per trial with equal probabilities. A probe stimulus subsequently appeared, consisting of two different numbers, each from 1 to 9, appearing next to each other. Either the left or right number (randomized per trial) had been presented in the trial’s memory set; the other number had not. Participants had to press a left (“F”) or right (“J”) response key to indicate which of the numbers was in the memory set. There was an inter-trial interval of 500 ms.

There were two variants of the task, which differed in terms of which distractors could occur during the maintenance period: a Baseline and a Full variant. In the Baseline task, there were two distractor types, which were equally likely to occur. Null distractors consisted only of a fixation cross. Neutral Face distractors consisted of one of a set of 11 male computer generated faces from the BESST (Thoma, Soria Bauser, & Suchan, 2013) with a neutral expression. The distractors were onscreen during the full maintenance period. This task consisted of three blocks of 24 trials each. The Baseline task was included in order to be able to consider effects of the neutral faces as natural distractor stimuli, i.e., without their role as cues for Attack stimuli as in the Full task described below.

In the Full task, a third distractor type, Attack, was introduced. Attack distractors initially started as Neutral Face distractors for the first 600 ms of the maintenance period. After this time, the expression was changed to anger and the size of the face was increased by 50%, creating a zoom-in effect. These changes occurred at the same time and instantaneously. The angry and zoomed-in face remained on-screen for the remainder of the maintenance period, i.e., for an additional 200 ms, 600 ms, or 1200 ms interval after the change. The Full task consisted of seven blocks of 24 trials each.

### Procedure

Participants first filled in questionnaires (demographics, Buss-Perry Aggression Questionnaire, PHQ-9, TSQ, STAI-6 and ad-hoc questions); please see the Appendix for further details and purely exploratory analyses involving questionnaires data. Subsequently they performed the Baseline and Full Emotional Sternberg Tasks. Following these tasks, two further tasks were performed that were part of different studies.

### Statistical Analyses

Preprocessing steps consisted of the removal of trials likely to deviate from normal task performance: the first four trials of the Baseline task, the first trial per block, and trials with very long RTs above 2500 ms. For the Full task the first block was removed, as this block was considered a learning block in which subjects experienced Attacks for the first time.

Within-subject Repeated Measures ANOVAs with Greenhouse-Geisser correction were used to analyze effects of Interval (the 200, 600 or 1200 ms following the initial 600 ms period) and Distractor Type (Null, Neutral Face, Attack). Effects were tested on median RT and mean accuracy. We used median RT to reduce the influence of outliers, without having to specify somewhat arbitrary criteria for the rejection of fast and slow trials, which would be necessary when using the mean. Interactions were probed using simple tests of effects using pairwise *t*-tests.

The original data are available on request.

## Results

Figures 2a and 2b shows the RT data for the VAEST. In the Baseline task, within-subject analyses showed an effect of Distractor Type, *F*(1, 58) = 24, *p* < .001, η^2^_p_ = 0.29, due to slower responses following Neutral Face than Null distractors. In the Full task, effects were found of Distractor Type, *F*(2, 116) = 23, *p* < .001, η^2^_p_ = 0.29, Duration, *F*(2, 116) = 7.4, *p* = 0.0013, η^2^_p_ = 0.11, and Distractor Type by Interval, *F*(4, 232) = 3.2, *p* = 0.017, η^2^_p_ = 0.052. All three distractor types significantly differed from each other (*p* < .001), Neutral Face being slowest, followed by Attack, followed by Null. The shortest interval was slower than the longer intervals (*p* = .013). These effects were modulated by the interaction, as clearly visible in the Figure as the decrease in interference over time for the Attack trials. On Attack trials only, the shortest interval (200 ms post-attack) led to slower responses (*p* = .0011) than the longer intervals (600 ms and 1200 ms post-attack).

**Figure 2a:**
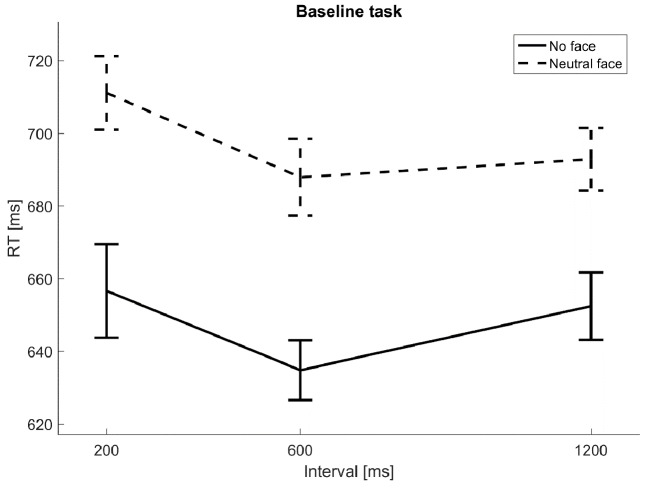
Reaction times: A. baseline task.

**Figure 2b:**
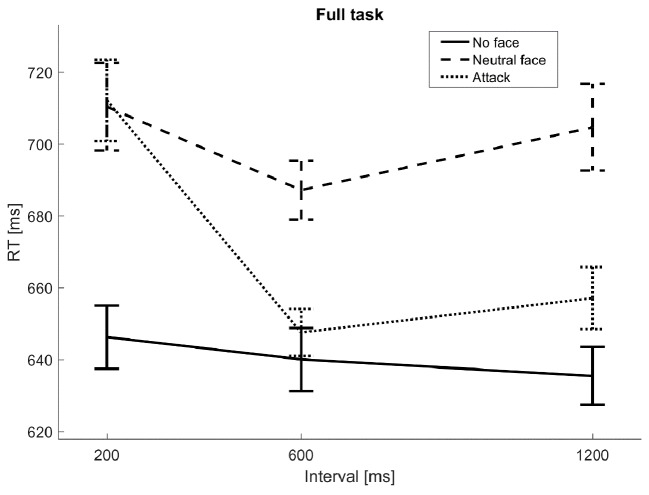
Reaction times: B. Full task. *Note*. The figure shows reaction time for the Baseline task (A) and Full task (B). Lines show mean values, and vertical bars show standard errors after correction for between-subject variability (as tests involved within-subject effects). The x-axis shows the interval between the 600 ms timepoint of maintenance period, when attacks could occur, and the presentation of the probe stimulus. In the Baseline task, the presentation of a Neutral face can be seen to cause slower RTs at all intervals. In the Full task, this effect remains. RTs on Attack trials are similar to RTs on Neutral Face trials at the shortest interval, but then strongly decrease, dropping close to the level of trials on which no face was presented as distractor.

No within-subject effects were found for accuracy, which was generally high (0.93 proportion correct in the Baseline task, 0.91 in the Full task).

## Discussion

In the current study we investigated interference effects in an emotional Sternberg task in which virtual attacks could occur, the VAEST. The primary question was whether virtual attacks would result in increased interference due to disruption of cognitive resources, or reduced interference due to the termination of a freeze state.

A basic result was that reaction times increased when showing a neutral face versus when no face was presented, even before any attacks had been shown. This can be contrasted to effects in a previous study (Gladwin, 2017), in which the presentation of the same computer-generated faces was facilitative, speeding responses. In the previous study, the task involved a simple speeded choice task, with a clear stimulus-response mapping involving left and right arrows mapped, respectively, to the left and right response keys. Thus, the effect of the facial distractor stimuli depends on task features. Even though the task in the current study was easy, it did require responses to be dependent on information held in working memory, as opposed to immediately available stimulus features. This difference appears likely to have induced a slowing versus facilitative effect on RTs. An interesting implication of these conflicting results is that while effects of emotional distractors are usually considered to reflect automatic, involuntary processes, they nevertheless depend on task-related factors (Wells & Matthews, 1996).

The primary question involved the effect of the occurrence of the virtual attack in the Full task. This clearly had a facilitative effect. The RT slowing induced by the neutral face was strongly decreased when the attack occurred, as long as sufficient time was provided between the attack and the probe. This would not be expected if interference can be explained by emotionally salient stimuli capturing processing resources. The angry expression has previously been shown to evoke stronger emotional responses than the neutral expression (Gladwin, 2017), and the sudden zoom-in would also be expected to evoke an emotional response (Montoya et al., 2015). The pattern of results has better agreement with the hypothesis that attacks serve to release participants from an inhibited state of freezing. That is: the neutral face evokes a freeze state, in which the expression of prepared actions is inhibited, which causes slowing on responses to probe stimuli. However, if an attack actually occurs, this serves as a trigger for ending the freeze state (Gladwin et al., 2016; Roelofs, 2017), which removed the slowing effect.

A limitation of the current study is that the neutral face distractor was already a very effective “natural” distractor in the Baseline task. Most of its effects cannot therefore be interpreted purely within the experimental context, in terms of anticipation of the virtual attack. It would be interesting for future work to use visually neutral cues to predict attacks. Another interesting line of research could be to further investigate the Attack stimulus. In the current study, this was a combination of a zoom-in effect and a change of expression from neutral to angry. Thus, the current results cannot determine which of these stimulus features was necessary or sufficient to cause effects, or which other stimuli could serve as effective Attacks or freeze-terminating stimuli. A methodological limitation is that only 200, 600 and 1200 ms post-attack intervals were used. As the major drop in slowing occurred from the 200 to 600 ms interval, future work should sample this region more extensively. Another methodological limitation is that the working memory aspect of the task was very simple, and it could be of interest to compare the current results with those found using a more taxing or complex task. Finally, our interpretation of results in terms of freeze is based on the fit of the pattern of behavioral results with previous work on freeze. However, psychophysiological data - such as body sway, heart rate, and EMG - are needed to more directly measure whether freeze occurred and how it was related to effects.

In conclusion, virtual attacks in an emotional Sternberg task were found to strongly reduce response slowing due to an initial distractor. This fits with a “freeze-release” model, in which the virtual attack triggers the end of an inhibited state. The results may be of interest for further research, in particular by raising the question whether slowing effects are due to interference with cognitive processing, versus due to a reversible inhibitory state. Finally, the interference effect could be of interest as a target for cognitive-emotional conditioning methods such as Cognitive Bias Modification. The VAEST could be converted to a training task in the same way as, e.g., approach-avoidance tasks (Wiers, Eberl, Rinck, Becker, & Lindenmeyer, 2011), to the aim of downregulating or improving the ability to release freezing responses.

## Appendix: Exploratory Correlational Analyses

A secondary aim of the study was to provide purely exploratory correlations between task effects and individual differences in subclinical symptoms of mental health disorders. The goal of these secondary analyses was solely to provide indications that could be useful for future confirmatory studies: it is important to note that this correlational aspect of the current paper does not attempt to control for multiple testing in order to reach statistical significance for each test. The goal is to aid in the generation of specific a priori hypotheses for future study, not to provide relatively conclusive statistical evidence.

Nevertheless, we felt that including these analyses could provide a helpful first step in connecting disorders to effects on the VAEST. Such connections seem likely from the dual-process perspective: certain mental health disorders could occur when automatic attentional processes select harmful information or inhibit helpful information, and when reflective processes are not evoked when needed to correct maladaptive impulses when this pattern of information is used for response selection (Gladwin, Figner, Crone, & Wiers, 2011; Pessoa, 2009). For example, in Attentional Control Theory, anxiety is posited to reduce attentional control and assign cognitive resources to threat-related stimuli (Eysenck & Derakshan, 2011; Eysenck, Derakshan, Santos, & Calvo, 2007). Processes underlying various mental health disorders could thus be expected to increase interference, as has indeed been found in a wide range of literature. Aggression and violence are associated with slowing effects on emotional Stroop tasks involving threat-related stimuli (Brugman et al., 2016; Chan, Raine, & Lee, 2010; Domes, Mense, Vohs, & Habermeyer, 2013). Individuals suffering from post-traumatic stress disorder (PTSD) also show increased interference to trauma-related stimuli (Aupperle, Melrose, Stein, & Paulus, 2012), e.g., in combat veterans with PTSD after deployment in Iraq to combat-related words (Ashley, Honzel, Larsen, Justus, & Swick, 2013) and in Vietnam veterans with PTSD to pictures related to Vietnam (Chemtob et al., 1999). Depression is associated with increased interference on depression-related words such as “hopeless” (Gotlib & McCann, 1984; Mathews & MacLeod, 1994; Williams & Nulty, 1986). Finally, anxiety is associated with increased interference by threat-related words (MacLeod, Mathews, & Tata, 1986; McNally, 1995; Phaf & Kan, 2007). We therefore tested whether subclinical symptoms in the current sample, involving aggression, trauma, depression and anxiety, were associated with increased interference effects on the VAEST.

In line with the above literature, a number of questionnaires were used that were considered of potential interest for future studies. The questionnaires concerned aggression, depression, psychological trauma and state anxiety and were filled in online, via custom HTML, PHP and Javascript. All items had to be answered to progress. For aggression, the Buss-Perry Aggression Questionnaire (Buss & Perry, 1992), was used, which provides four subscales: physical aggression, verbal aggression, anger, and hostility. Subscales are the sum of the (if necessary, reverse-scored) respective set of items, each scored on a 1 through 7 Likert scale. Example items are, for the subscale physical aggression, “Once in a while I can't control the urge to strike another person”; for verbal aggression, “I can't help getting into arguments when people disagree with me.”; for anger, “I sometimes feel like a powder keg ready to explode.”; and for hostility, “I am suspicious of overly friendly strangers.”. The Buss-Perry scales have been found to have adequate reliability and validity (Bernstein & Gesn, 1997; Buss & Perry, 1992; Harris, 1997). For depression, the depression module of the Patient Health Questionnaire, the PHQ-9, was used, which provides a brief but reliable and valid measure of depression (Cameron, Crawford, Lawton, & Reid, 2008; Kroenke, Spitzer, & Williams, 2001; Titov et al., 2011). Responses required indicating to which extent a certain problem had been experienced over the last two weeks, on a Likert scale from 1 to 4, represented by response categories "Not at all", "Several days", "More than half the days", and "Nearly every day". The score is the sum over all items. An example item is “Feeling down, depressed, or hopeless”. The Trauma Screening Questionnaire, TSQ (Brewin et al., 2002), was used to measure post-traumatic stress symptoms. The TSQ score is the sum of ten 0 or 1 coded items reflecting a Yes or No response to having experienced a given reaction at least twice in the last week. An example item is “Upsetting thoughts or memories about the event that have come into your mind against your will”. The TSQ has been shown to provide a close approximation to gold standard diagnostic interviews for post-traumatic stress disorder, using a cut-off score of 6, and has adequate reliability and validity (de Bont et al., 2015; Walters, Bisson, & Shepherd, 2007). Finally, the six-question Spielberger State-Trait Anxiety Inventory, STAI-6 (Marteau & Bekker, 1992), was used to measure state anxiety. The STAI score is the sum over six items about how the participant feels, each scored on a Likert scale from 1 to 4 (for positive items) and -1 to -4 (for negative items), with response categories labelled "Not at all (1)", "Somewhat (2)", "Moderately (3)", and "Very much (4)". An example item is “I feel calm”. The STAI-6 has been found to have adequate reliability and validity comparable to the full STAI (Marteau & Bekker, 1992).

In the exploratory secondary analyses, Pearson's correlations were calculated between various contrast scores involving task performance and questionnaire scores. Contrast scores were, first, Neutral Face versus Null, for interference effects due to the appearance of the neutral face versus no distractor during the maintenance period. This contrast was calculated both for the Baseline and for the Full task. Further, there was the contrast Neutral Face versus Attack, reflecting effects related to the virtual attack versus the absence of a virtual attack occurring. Finally, an Anticipation contrast was calculated as the difference between the contrasts for Neutral Face versus Null in the Full versus Baseline tasks. This contrast was interpreted as representing effects caused by the anticipation of the virtual attack in the Full version: there is then an additional contextual effect of presentation of a neutral face, which was absent when the neutral face was presented in the Baseline task when no attacks could yet occur.

Table A.1 shows descriptive statistics of the questionnaires.

**Table A.1:** Descriptive Statistics

Variable	*M*	*SD*	*n*
BP: Physical Aggression	21.60	10.20	58
BP: Verbal Aggression	17.50	7.08	58
BP: Anger	16.50	5.78	58
BP: Hostility	19.30	7.57	58
PHQ9	13.80	3.74	59
TSQ: Total	2.45	2.27	58
TSQ: Cutoff6	0.12	0.33	58
STAI-6	-4.31	3.60	59

*Note*. Means, standard deviations and sample size for questionnaire data. For some questionnaires, data for one subject was lost due to technical issues. The variables are the Buss-Perry Aggression Questionnaire subscales, the PHQ9 scale for depression, the TSQ questionnaire, and the six-item STAI scale for state anxiety. Two values were included for the TSQ: the total score, which was the number of endorsed items concerning re-experiencing and arousal symptoms, and a cut-off score. The cut-off score was 0 if the total score was below 6, and 1 otherwise.

Results of the correlation analyses are shown in Table A.2a and Table A.2b. On RT, the only effect involved depression: higher PHQ-9 scores were associated with slower responses specifically on the Neutral Face trials in the Full task. This effect was found for the contrast between Neutral Face trials and Null trials, the contrast between Neutral Face trials and Attack trials, and the Anticipation contrast. Thus, depressive symptoms appeared to lead to increased slowing when an Attack was anticipated and did not occur.

On accuracy, a number of effects were found in which symptoms were related to lower accuracy on Neutral Face trials. Anger and the binary score for reaching the cut-off TSQ-score for PTSD were associated with lower values on the Neutral-Null (Baseline) contrast, while depression and total symptom score for PTSD were associated with lower values on the Neutral-Null (Full) contrast. However, physical aggression was associated with higher values on the Neutral-Null (Full) contrast. The Anticipation contrast (Neutral-Null for the Full versus Baseline task) was positively related to physical aggression, verbal aggression, and anger. That is: these aspects of aggression all resulted in relatively high accuracy when anticipating an Attack.

**Table A.2a:** Significant Correlations Involving Contrast Scores and Questionnaire Data: A. Reaction Time

Scale	Neutral-Null (Baseline)	Neutral-Null (Full)	Neutral-Attack	Anticipation
BP: Physical Aggression				
BP: Verbal Aggression				
BP: Anger				
BP: Hostility				
PHQ9		.29 (1200 ms)	.26 (1200 ms)	.33 (1200 ms)
TSQ: Total				
TSQ: Cut-off				
STAI-6				

**Table A.2b:** Significant Correlations Involving Contrast Scores and Questionnaire Data: B. Accuracy

Scale	Neutral-Null (Baseline)	Neutral-Null (Full)	Neutral-Attack	Anticipation
BP: Physical Aggression		.28 (200 ms)		.33 (200 ms)
BP: Verbal Aggression				.30 (200 ms)
BP: Anger	-.29 (200 ms)			.29 (200 ms)
BP: Hostility				
PHQ9			-.28 (1200 ms)	
TSQ: Total			-.31 (1200 ms)	
TSQ: Cut-off	-.26 (200 ms)			
STAI-6				-.28 (600 ms)

*Note*. Correlations between contrast scores derived from the VAEST and questionnaire data. The contrast scores were calculated as follows, for RT and accuracy in Tables A.2a and A.2b respectively. The contrast “Neutral - Null (Baseline)” is the difference score for Neutral Face trials minus Null trials in the Baseline task. The contrast “Neutral - Null (Full)” is the difference score for Neutral Face trials minus Null trials in the Full task. The contrast “Neutral - Attack” is the difference score for Neutral Face trials minus Attack trials in the Full task. Finally, the “Anticipation” score is the double-difference score between Neutral - Null (Full) and Neutral - Null (Baseline). This reflects effects of knowledge of a possible attack, as in the Full task neutral faces predict a possible attack. The questionnaires are as in Table A.1. Note that due to the varying Cue-Stimulus Intervals, each contrast can be calculated for each of the three possible intervals. Intervals for which the reported contrast scores were calculated are given in brackets behind the correlations: the durations are the time of a possible attack (600 ms into the maintenance interval) to the presentation of the probe (after a further 200, 600 or 1200 ms).

Thus, despite the preliminary and exploratory nature of these analyses, there were some consistent patterns. Depressive symptoms were related to increases in three contrast scores, all of which could be interpreted as slowing due to anticipation of the virtual attack. This agrees with literature showing automatic cognitive biases in depression (Johnstone, van Reekum, Urry, Kalin, & Davidson, 2007; MacLeod & Rutherford, 1998) and suggests a potential target for cognitive bias modification (MacLeod, Koster, & Fox, 2009; Wiers, Gladwin, Hofmann, Salemink, & Ridderinkhof, 2013). Another consistent effect involved three subscales of the Buss-Perry Aggression Questionnaire (physical aggression, verbal aggression, and anger), all of which were related to an improvement in accuracy in the context of a potential attack. Aggression is known to involve hypervigilance and arousal (Heesink, Kleber, et al., 2017; Jaworska et al., 2012), and increased arousal may have improved performance in this relatively simple task. Alternatively, aggressive individuals may be more used to interpreting neutral faces as potentially aggressive (Dodge et al., 2015). Various other effects were found, most of which involved reduced accuracy for only one of the contrasts involving freezing on neutral faces, with increasing psychological trauma and anxiety. Such effects agree with results and theories on trauma, in which effects of trauma on emotionally important schemata render individuals more vulnerable to automatic interference by emotional stimuli (Beck, Freeman, Shipherd, Hamblen, & Lackner, 2001; Bryant & Harvey, 1995; Chemtob et al., 1999; McNally, Kaspi, Riemann, & Zeitlin, 1990; Pineles, Shipherd, Mostoufi, Abramovitz, & Yovel, 2009; Pineles, Shipherd, Welch, & Yovel, 2007). Future studies are needed to further elucidate relationships between interference effects and problems related to the integration of negative information into schemata. A potentially important focus could be disentangling aspects of repetitive negative thinking and intrusive thoughts (Ehring et al., 2011; Ehring, Frank, & Ehlers, 2008; Horowitz, Wilner, & Alvarez, 1979; McEvoy et al., 2017). However, it must be acknowledged that any interpretation is limited due to the exploration of effects over many tests and the relatively small student sample. The results are hoped to provide clear directions and a basis for methodological choices (e.g., contrast score definitions) for future work, e.g., comparing clinical populations.
